# Journal of Circadian Rhythms: 10th anniversary

**DOI:** 10.1186/1740-3391-11-1

**Published:** 2013-01-04

**Authors:** Roberto Refinetti

**Affiliations:** 1 Circadian Rhythm Laboratory, University of South Carolina, Walterboro, SC, 29488, USA

## 

The year in which this editorial is being published (2013) marks the 10th anniversary of the launch of the *Journal of Circadian Rhythms*. Back in 2003, I wrote that “various researchers in the United States, Europe, and developing countries believe that scientific publishing in the 21st century will be characterized by open access to (peer-reviewed) articles, freely and universally available on-line to readers worldwide”. Since the launch of the journal, electronic publishing has gone from a rarity to commonplace – and, although there are still many subscription based journals, the success of open access and the “author pays” model is becoming more and more evident. Many large publishers today include fully open access titles within their journal portfolios, and submissions to BioMed Central (the publisher of the *Journal of Circadian Rhythms*) have increased from under 5000 in 2003 to over 40,000 in 2011 [1].

In recognition of the fact that new research must not lose sight of the fundamentals established in the past, I invited Professor Franz Halberg to write the very first article published in the journal [2]. Halberg, the creator of the term *circadian* and foremost advocate of the discipline of chronobiology, had been a leading figure in biological rhythm research for over 50 years and is still very active today, a few years shy of his 100th birthday. The article recounted his trajectory from the discovery of circadian rhythms to the development of the notion of the chronome, a notion that he continues to advocate with great enthusiasm through numerous theoretical articles and internationally collaborative medical research. His article elicited great interest in readers, who have so far downloaded it over 40,000 times (the highest number of downloads of any article published in the journal to date).

Many articles have been published since the publication of Halberg’s inaugural article. Of all articles published in the *Journal of Circadian Rhythms* to date, 10% were literature reviews, 57% were basic-science research articles, and 33% were clinical/applied articles. The breakdown by research subjects was as follows: 2% plants and bacteria, 6% invertebrate animals, 1% birds, 32% rodents, 49% humans, and 10% other mammals. The topics of study reported in these articles were, not surprisingly, consistent with the journal’s mission. They covered circadian and nycthemeral (daily) rhythms in living organisms at all levels of biological organization (molecular, cellular, organic, organismal, and populational) as well as daily rhythms in environmental factors that directly affect circadian rhythms.

Highlighting some articles but not others may give the impression that some articles are more appreciated by the editor than others, but the statistics of reader demand make the task comfortably objective. Seven articles have been accessed more than 15,000 times and deserve at least brief mention here. As indicated above, Halberg’s inaugural article was accessed more times than any other article. Two of the other six articles are literature reviews, which is consistent with the fact that review articles tend to receive more citations than regular research reports [3]. One of these articles reviewed the literature on neurotransmitters involved in neural communication within the mammalian master circadian clock in the suprachiasmatic nuclei [4], whereas the other reviewed the literature that investigates the adaptive significance of circadian rhythms and how it may have affected the evolution of organisms on Earth [5].

The most highly accessed research article so far is my own brief study on nycthemeral variation in the frequency of sexual contacts in humans [6]. Counter-intuitively, the article indicated that the time of day at which people tend to have sex (bedtime, on average) is determined not by romantic feelings but by mere convenience (as the two people are together in bed in preparation for sleep). Another highly accessed article is one dealing with the impact of day-night inversion on the strength of the circadian organization of behavior [7]. The authors conducted the study in both shift-working nurses and laboratory rats. Rotating-shift nurses and “jet-lagged” rats had weaker circadian organization of behavior than nurses and rats exposed to regular light–dark patterns.

Of the other two highly-accessed research articles, one dealt with the effect of illumination color on the wellbeing and work performance of office workers [8]. The authors found that a light source emitting more blue light than standard fluorescent lighting can improve the wellbeing and productivity of office workers. The other article documented synchronization of circadian activity rhythms by a light–dark cycle in visually blind marmoset monkeys [9], thus confirming in a non-human primate the finding in humans that the circadian system of visually blind individuals can “see” light [10].

These seven articles just described are, of course, only a small sample of articles published during the first 10 years of the journal. Readers are encouraged to browse the list of articles and read them all, as they are all open-access. Prospective authors are encouraged to submit manuscripts for potential publication. The journal welcomes research in all areas of circadian rhythm biology – from the physiological aspects to novel advances in the fields of neuroscience, molecular biology and genetics. Because 91% of the articles published in the journal have dealt with mammalian subjects, submission of articles dealing with non-mammalian species is especially encouraged, although we will, of course, continue to welcome articles dealing with circadian rhythms in any life form (as well as those dealing with daily rhythms of environmental variables that affect living organisms).

This year we will be publishing a thematic series on the Numerical Analysis of Circadian Rhythms, which will include a number of invited reviews from leaders in the field. Further research and review articles are also welcome, so please use the online submission system to submit to the series. Prospective authors and readers are reminded that, as it has done for the past 10 years, the *Journal of Circadian Rhythms* will be on-line 24 hours a day, 7 days a week, bringing the latest research developments in the study of circadian rhythms promptly and freely to readers worldwide.
